# Supplementary material for: Hesitancy in COVID-19 Vaccine Uptake and Its Correlated Factors Using Multi-Theory Model among Adult Women: A Cross-Sectional Study in Three States of Somalia
Source: Vaccines (Basel). 2023 Sep 14;11(9):1489. doi: 10.3390/vaccines11091489 (PMC10534331; doi:10.3390/vaccines11091489)
Supplement: Supplementary file 1 [file vaccines-11-01489-s001.zip › vaccines-2216409-supplementary.pdf]

Supplementary Material

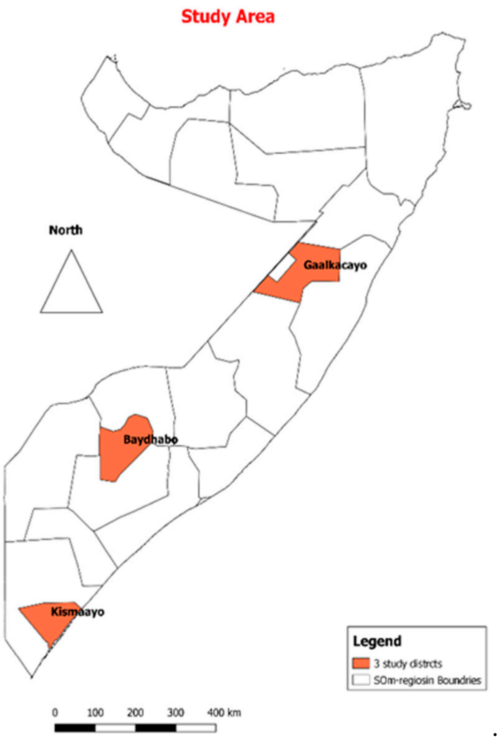

Figure S1. Study location: Baidao, Kismayo, and Galkayo districts in Somalia.

Table S1. Sampled respondents.

State	Region (district)	Villages	IDPs or Host Co	Target Population	Respondents
**Southwest State**	**Bay (Baidao)**	Isha	Host Co	89	77
		Berdaale	Host Co	89	77
		Horseed	Host Co	89	77
		Hanano 2 IDP	IDP	59	51
		11janayo IDP	IDP	59	51
**Puntland State**	**Mudug (Galkayo)**	New Garsoor laanta 4	Host Co	89	77
		Siinay	Host Co	89	77
		Israc Buulo-dooro	Host Co	89	77
		Ayax IDP	IDP	59	51
		Liban 1 and 2	IDP	59	51
**Jubbaland State**	**Lower Jubba (Kismayo)**	Farjano	Host Co	89	77
		Midnimo	Host Co	89	77
		Guulwade	Host Co	89	77
		Galbeed IDPs	IDP	59	51
		Fanole IDPs	IDP	59	51
Total				**1155**	**999**
**Response rate = 86.5%**					

Table S2: Response Rate Calculation

**Response Rate calculation**		
1	Complete interview	999
P	Partial interview	81
R	Refusal and break-off	47
NC	non-contact	9
O	Other	12
UH	Unknown if household/occupied HU	1
UO	Unknown, other	6
Total		156

RR=999/ (999+81+47+9+12+1+6) = 86.5%.

Response Rate Calculation

The response rate (RR) is the number of complete interviews with reporting units divided by the number of eligible reporting units in the sample (Montgomery, Dennis, & Ganesh, 2016).

The **non-response** rate is defined as the percentage of all potentially eligible units (or elements) that do not have responses to at least a certain proportion of the items in a survey questionnaire (Shaw, Bednall, & Hall, 2002).

The following formula was used to calculate the response rate.

$$\mathbf{RR1} = \frac{\mathbf{I}}{(\mathbf{I} + \mathbf{P}) + (\mathbf{R} + \mathbf{NC} + \mathbf{O}) + (\mathbf{UH} + \mathbf{UO})}$$

Where, RR1 = Response rate
I = Complete interview
P = Partial interview
R = Refusal and break-off
NC = non-contact
O = Other
UH = Unknown if household/occupied HU
UO = Unknown, other

The details of these quantities for our study are given in the following:

I and P=The in-person household survey was conducted in which housing units are sampled from an address-based sampling frame of 15 selected villages using systematic sampling technique. We consider less than 50% of all applicable questions answered (with other than a refusal or no answer) equals break-off, 50%-80% equals partial, and more that 80% equals complete. We found complete answered from 999 participants (i.e., I=999). We found 81 of the participants did not complete the questionnaire (i.e., P=81).

R= Refusals and breakoffs consist of cases in which some contact has been made with the housing unit and a responsible household member has

declined to do the interview, or an initiated interview results in a terminal break-off (i.e., R=47).

NC= non-contacts in in-person household surveys consist of three types: a) unable to gain access to the building, b) no one reached at housing unit, and c) respondent away or unavailable (i.e., NC=9).

O= Other cases represent instances in which the respondent is/was eligible and did not refuse the interview, but no interview is obtainable because of a) the respondent is physically and/or mentally unable to do an interview; b) miscellaneous other reasons. We did not face any language problem to exclude participants. (i.e., O=12).

UH= Cases of unknown eligibility and no interview include situations in which it is not known if an eligible housing unit exists and those in which a housing unit exists (i.e., UH=1).

UO= Not eligible cases for in-person household surveys include a) out-of-sample housing units; b) not-a-housing unit; c) vacant housing units; d) housing units with no eligible respondent; and e) situations in which quotas have been filled. In a systematic sampling technique, we found a total of 6 households without any adult respondents during the interview (UO=6).

Thus, the response rate is, RR=999/ (999+81+47+9+12+1+6) = 86.5%

Table S3. multi-nominal logit analysis of determinants of intention to receive vaccine, undecided used as base category.

	Intended to receive the vaccine				Not intended to receive the vaccine			
	**Coeff.**	**Std.**	**z**	**P>z**	**Coeff.**	**Std.**	**z**	**P>z**
Household head								
Female (and..)	0.383	0.267	-1.380	0.168	0.443	0.441	-0.820	0.413
Education	0.263	0.182	-1.930	0.054	0.679	0.665	-0.390	0.693
Informal education	0.384	0.262	-1.400	0.161	0.945	0.919	-0.060	0.953
No education	0.641	0.449	-0.640	0.525	1.031	1.034	0.030	0.976
Primary								
Secondary	0.377	0.082	-4.460	0.000	0.619	0.186	-1.600	0.110
Income category	0.299	0.079	-4.540	0.000	0.518	0.194	-1.760	0.079
$50-150	0.327	0.140	-2.620	0.009	1.305	0.693	0.500	0.615
$150-250	0.202	0.108	-3.000	0.003	1.930	1.185	1.070	0.285
$250-350								
Above $350	1.389	0.394	1.160	0.246	1.874	0.844	1.390	0.163
Marital status	0.466	0.197	-1.810	0.071	0.698	0.397	-0.630	0.527
Married	0.913	0.297	-0.280	0.781	2.109	0.993	1.590	0.113
Single	1.023	0.032	0.740	0.461	0.923	0.040	-1.860	0.062
Widowed								
Number of children	1.457	0.293	1.870	0.061	1.053	0.319	0.170	0.864
Type of residence								
IDP	**0.380**	**0.090**	**-4.080**	**0.000**	0.600	0.218	-1.400	0.160
District	**0.220**	**0.049**	**-6.760**	**0.000**	0.318	0.093	-3.930	0.000
Galkayo	**5.305**	**1.122**	**7.890**	**0.000**	0.336	0.104	-3.520	0.000
Kismayo								

Trust in covid-19 vaccine	1.045	0.182	0.250	0.800	1.340	0.326	1.200	0.229
Previously took vaccine for other disease								
No	1.039	0.257	0.150	0.877	1.325	0.489	0.760	0.446
Status	1.183	0.290	0.680	0.494	1.569	0.547	1.290	0.196
Lactating	0.731	0.215	-1.060	0.288	1.920	0.795	1.570	0.115
None								
Pregnant	0.976	0.174	-0.140	0.891	1.181	0.307	0.640	0.522
Heard of bad information about vaccine								
Yes	1.102	0.198	0.540	0.591	1.371	0.345	1.250	0.210
Do you know anyone vaccinated								
Yes	1.905	0.405	3.030	0.002	0.948	0.250	-0.200	0.838
Do you have information about vaccine	1.341	0.290	1.360	0.175	0.471	0.127	-2.800	0.005
b. Yes,	0.992	0.846	-0.010	0.993	0.615	0.731	-0.410	0.682
Do you trust your healthcare provider (yes)	0.383	0.267	-1.380	0.168	0.443	0.441	-0.820	0.413
_cons	0.263	0.182	-1.930	0.054	0.679	0.665	-0.390	0.693

Reference:

Montgomery, R., Dennis, J. M., & Ganesh, N. (2016). Response rate calculation methodology for recruitment of a two-phase probability-based panel: The case of AmeriSpeak. *University of Chicago National Opinion Research Center white Paper*.

Shaw, M., Bednall, D., & Hall, J. (2002). A proposal for a comprehensive response-rate measure (CRRM) for survey research. *Journal of Marketing Management, 18*(5-6), 533-554.
